# Prediction of MicroRNA-Disease Associations Based on Social Network Analysis Methods

**DOI:** 10.1155/2015/810514

**Published:** 2015-07-26

**Authors:** Quan Zou, Jinjin Li, Qingqi Hong, Ziyu Lin, Yun Wu, Hua Shi, Ying Ju

**Affiliations:** ^1^School of Information Science and Technology, Xiamen University, Xiamen 361005, China; ^2^School of Computer Science and Technology, Tianjin University, Tianjin 300072, China; ^3^Software School, Xiamen University, Xiamen 361005, China; ^4^College of Computer and Information Engineering, Xiamen University of Technology, Xiamen 361024, China

## Abstract

MicroRNAs constitute an important class of noncoding, single-stranded, ~22 nucleotide long RNA molecules encoded by endogenous genes. They play an important role in regulating gene transcription and the regulation of normal development. MicroRNAs can be associated with disease; however, only a few microRNA-disease associations have been confirmed by traditional experimental approaches. We introduce two methods to predict microRNA-disease association. The first method, KATZ, focuses on integrating the social network analysis method with machine learning and is based on networks derived from known microRNA-disease associations, disease-disease associations, and microRNA-microRNA associations. The other method, CATAPULT, is a supervised machine learning method. We applied the two methods to 242 known microRNA-disease associations and evaluated their performance using leave-one-out cross-validation and 3-fold cross-validation. Experiments proved that our methods outperformed the state-of-the-art methods.

## 1. Introduction

MicroRNAs constitute a class of non-protein-coding small RNAs, 20 to 25 nucleotides long, that bind to the 3′ untranslated region of target mRNAs to regulate mRNA turnover and translation. There are many biological processes, which are regulated by microRNAs, such as development, differentiation, apoptosis, and diseases [1–3]. Many studies have found that microRNAs play an important role in cellular signaling networks [4], tissue development, [5–7] and cell growth [8]. They are also associated with various diseases [9, 10], including breast cancer [11, 12], lung cancer [13, 14], cardiomyopathy [15], and cell lymphoma [16]. If the microRNA abnormality causes the disease, the abnormal microRNA and the disease are associated by the causal relationship. And the microRNA-disease association is what we aim to predict. Predicting microRNA-disease associations has emerged as an important strategy in understanding disease mechanisms [17]. For example, dysregulation of microRNAs can affect apoptosis signaling pathways and cell cycle regulation in cancer [18].

The importance of microRNA-disease association prediction has been appreciated for some time [19]. However, most of the techniques that have been developed to achieve this suffer several inherent weaknesses; in particular, traditional experimental approaches are time-consuming and expensive. It is necessary to employ the bioinformatics analysis, which could make use of databases and the potential inferences. For bioinformatics approaches, it is important to measure the functional similarities among microRNAs in order to construct networks based on functional similarity [20–24]. The construction of functional similarity networks for genes encoding proteins has produced significant results [25–32]; however, the methods used to analyze protein-encoding genes are not always adaptable to enable use with microRNAs because the correlation between the functional similarities of genes and gene sequences or expression similarities may not exist for microRNAs [5, 6, 33, 34]. MicroRNAs directly adjust the one-third of the human genes. The genes targeted by miRNAs identified are recognized from directed biological process. However, the previous published methods to find gene used bio-experiment or the characteristics of protein sequence. However, gene and miRNA identification is quite inefficient. Another issue is that there are not many validated associations between microRNAs and diseases. For studying microRNA-disease association, there are two well-known databases: the human microRNA-associated disease database (HMDD) and the miR2Disease database of differentially expressed MiRNAs in human cancers (dbDEMC). The data in HMDD and dbDEMC are manually collected and archived from publications [10, 21, 22, 35]. The last main challenge is that it is difficult to select negative samples as there are no verified negative microRNA-disease associations. It is therefore difficult to conduct biological experiments without such controls. Hence, it is necessary to develop effective computational methods to detect potential microRNA-disease associations.

To overcome the above challenges and to effectively predict associations, we explored the computational method KATZ [36] and the machine learning method CATAPULT [5, 6] to predict microRNA-disease associations. The two methods can succeed to overcome the challenges above. The highlight work is to discover unknown associations through known associations, including microRNA-microRNA associations, a small quantity of microRNA-disease associations, and disease-disease associations. Previous studies show that one or more mutations from the same functional module can give rise to diseases with overlapping clinical features [1, 37–39]. Biological experiments of human disease show that microRNAs causing similar diseases often interact with each other directly or indirectly [40–45]. Hence, we learn from the idea of social network. This is an integrated network composed of microRNA-microRNA association networks, known microRNA-disease association networks, and disease-disease association networks and is similar to social networks used to predict the relationship between two individuals [40, 46–49]. In this paper, we take full advantage of relationships among microRNAs and diseases to predict the association between microRNA and disease. Each predicted microRNA-disease association is denoted by a score. For each disease, we rank the microRNA on the basis of a score. For a disease, if a microRNA is ranked in the top *k*, the microRNA is expected to have a high probability of association with the disease [50, 51]. We show that KATZ and CATAPULT are superior to current methods by cross-validation. KATZ and CATAPULT are able to propose many potential associations, which is of great value for future studies.

## 2. Datasets

We used three types of data, microRNA-microRNA association, microRNA-disease association, and disease-disease association data. The microRNA-microRNA association dataset includes 271 microRNAs, and the association is denoted by a functional similarity score. The dataset was downloaded from http://www.webcitation.org/query.php [5, 6]. The disease-disease association dataset, including 5080 diseases, was downloaded from MimMiner [52], which provides a similarity score for each phenotype pair by text mining analysis of their phenotype descriptions in the Online Mendelian Inheritance in Man (OMIM) database [53]. The disease-disease similarity scores have been successfully used to predict or prioritize disease related genes [54, 55]. The microRNA-disease association dataset contained 271 microRNAs and 5080 diseases. Furthermore, there are 242 microRNA-disease associations. It means there are 242 nonzero elements in the matrix of microRNA-disease association. The microRNA-disease association dataset was downloaded from [56]. In addition, we verified that the 242 nonzero elements consisted of 99 microRNAs and 51 diseases. The details of the datasets are shown in Table 1.

**Figure 1:**
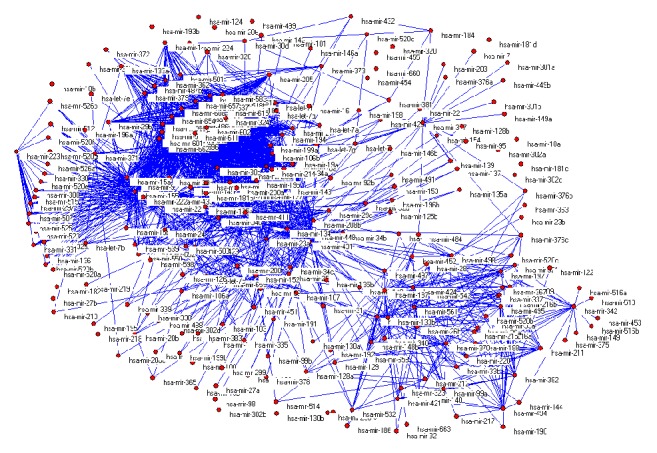
Bipartite graph of the microRNA-disease association network.

With the above datasets, we could construct a microRNA-microRNA network, a disease-disease network, and a microRNA-disease network using a bipartite graph. For example, Figure 1 denotes the bipartite graph of the microRNA-disease network. In the graph, the nodes denote microRNAs or diseases and the lines correspond to associations between microRNAs and diseases. If there is an association between a microRNA and a disease, there must be a line between the microRNA and the disease.

**Figure 2:**
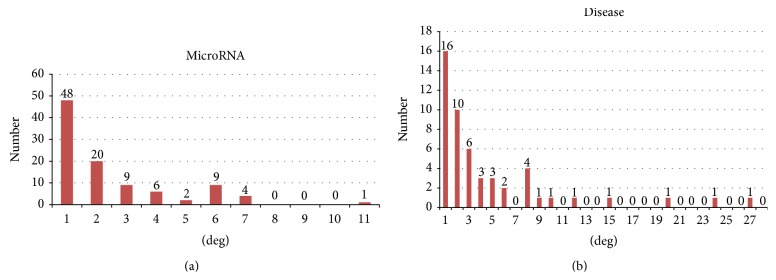
Degree distributions of microRNAs and diseases in the bipartite graph of the microRNA-disease association network.

The degree distributions of microRNAs and diseases in the bipartite graph of the microRNA-disease association network are illustrated in Figure 2. The microRNA degree is defined as the number of diseases that connect with a microRNA. In the same way, the disease degree is defined as the number of microRNAs that connect with a disease. The node degree can show the activeness or status of the node (microRNA or disease) in the entire network.

We propose to compare our methods with the previously described microRNA-based similarity inference (MBSI), phenotype-based similarity inference (PBSI), and network-consistency-based inference (NetCBI) methods [55]. Hence, we used the same datasets as them and we present Table 2 to clearly describe the statistical data for the bipartite graph of the microRNA-disease association network. Table 2 illustrates that there are few known microRNA-disease associations for a disease. For example, it should have 271∗5080 microRNA-disease associations, but known associations are only 242.

## 3. Methods and Algorithm

We introduce two different computational methods, which were presented by [36] to predict microRNA-disease associations. The first method, KATZ [57], has been shown to be successful at predicting links in a social network. When KATZ is applied to predict microRNA-disease associations, it uses the functional similarity score to denote the associations. KATZ computes the similarity score based on walks of different lengths between the microRNA and disease nodes. The second method, CATAPULT, is a supervised learning method. For the supervised learning method, features must be offered that are derived from hybrid walks through the microRNA-disease association network. However, CATAPULT is a transformation of a general supervised learning method. For the problem of microRNA-disease association, there are only positive examples and unlabeled examples, which CATAPULT is able to overcome. Algorithm part will detailedly present KATZ and CATAPULT.

### 3.1. KATZ

KATZ is similar to classical approaches, such as random walk [58], Prince [59], and CIPHER [60]. The essence of these approaches is a ranking algorithm. For example, the KATZ method computes the functional similarity score for microRNA-disease node pairs based on the microRNA-disease association network and ranking the diseases for a microRNA on the basis of the functional similarity score [57]. KATZ was successfully applied to predict social associations based on a social network [60]. Predicting microRNA-disease associations on the basis of a microRNA-disease association network is equivalent to predicting associations in a social network. KATZ results show that it can also adapt to predict associations between microRNAs and diseases.

**Figure 3:**
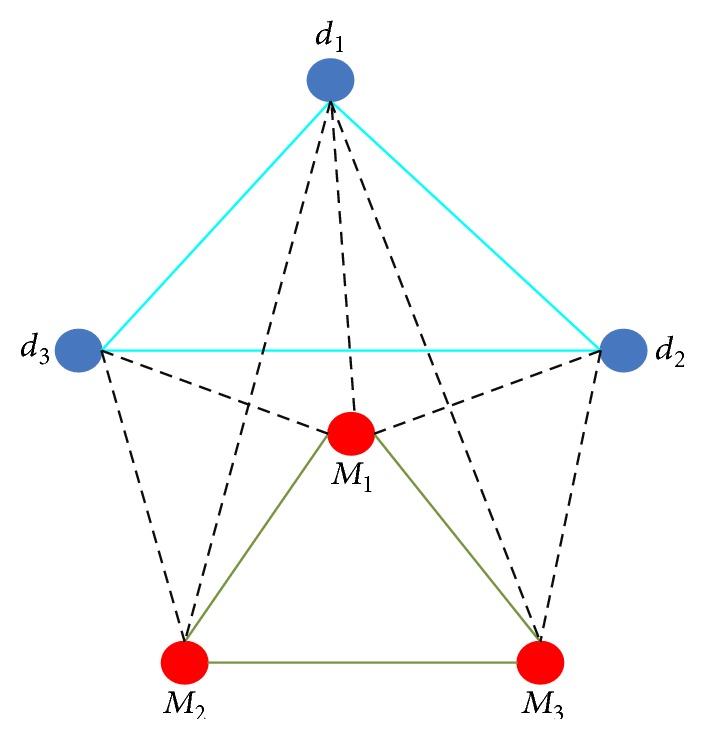
Unweighted, undirected graph.

For the known associations between microRNAs and diseases, we constructed an unweighted, undirected graph and derived a corresponding adjacency matrix of the graph. To vividly describe the method, we illustrate a simple unweighted, undirected graph, in Figure 3. Suppose the corresponding adjacency matrix of Figure 1 is *A*; the adjacency matrix *A* can be written with *A*_*ij*_ = 1, if microRNA node *i* and disease node *j* are connected, and *A*_*ij*_ = 0, if there is no line between microRNA node *i* and disease node *j*. However, there are not many direct lines linking microRNA and disease; therefore, it is difficult to denote the microRNA-disease association through the adjacency matrix *A*. Thus, we counted the number of walks of different lengths, which link microRNA node *i* and disease node *j* to signify the association between microRNA and disease. (*A* ^*l*^)_*ij*_ denotes the number of walks of length *l* that link node *i* and node *j*.

Next, we integrated different walks of different length to obtain a comprehensive association measure. We introduced a nonnegative coefficient *β*_*l*_, whose function is to control the contribution of different length walks. If *l*1 is larger than *l*2, *β*_*l*1_ is smaller than *β*_*l*2_. Suppose microRNA node *i* and disease node *j* are not connected in the unweighted, undirected graph; then *A*_*ij*_ = 0 and the microRNA *i* and disease *j* association can be computed through

(1) $$\begin{array}{c} S{\left(A\right)}_{ij}=\overset{k}{\underset{l=1}{\sum }}\beta^{l}{\left(A^{l}\right)}_{ij}. \end{array}$$

From formula (1), we can draw the conclusion that higher order paths contribute much less to microRNA-disease association. Formula (2) can process the entire unweighted, undirected graph:

(2) $$\begin{array}{c} S=\overset{k}{\underset{l=1}{\sum }}\beta_{l}A^{l}, \end{array}$$

where if *l* → *∞*, *β*_*l*_ → 0. In KATZ, if *β*_*l*_ is replaced by *β* ^*l*^, KATZ can be written as

(3) $$\begin{array}{c} S^{\text{katz}}=\sum_{l\geq 1}\beta^{l}A^{l}={\left(I-\beta A\right)}^{-1}-I, \end{array}$$

where *β* is chosen on the basis of *β* < 1/‖*A*‖^2^. For the choice of value *k*, the sum over infinitely many path lengths is not necessarily considered. According to the experimental results, small values of *k* (*k* = 3 or *k* = 4) obtain good performance in the task of recommending linked nodes. We have carried out the experiments for the other values of *k*. When *k* < 3, the experimental results are worse. However, for *k* > 4, the results are no better than *k* = 3 or 4. In addition, when *k* > 4 or bigger, the experimental time is much longer.

To use KATZ, we need a microRNA-disease association adjacent matrix *A*, which is the adjacent matrix of the microRNA-disease association network and is denoted as follows:

(4) $$\begin{array}{c} A=\left[\begin{array}{cc} G_{\text{MM}} & G_{\text{MD}}\\ G_{\text{MD}}^{T} & G_{\text{DD}} \end{array}\right], \end{array}$$

where *G*_MM_ is the adjacent matrix of the microRNA-microRNA association network, *G*_MD_ is the adjacent matrix of the microRNA-disease association network, and *G*_DD_ is the adjacent matrix of the disease-disease association network. We substituted the adjacent matrix *A* into formula (3) to obtain the association score matrix of microRNAs and diseases.

Setting *k* = 3, the correlation score matrix *S* ^KATZ^(*A*) denoting the association between microRNAs and diseases can be written as expression (5). Here we use KATZ with *k* = 3 to obtain the correlation score matrix. Consider

(5) SKatzA=βGMD+β2GMMGMD+GMDGDD+β3GMDGMDTGMD+GMM2GMDhhhh.+ GMMGMDGDD+GMDGDD2.

One of the advantages of  KATZ is that it can study human microRNA-disease association and association for other species. In  KATZ, this is achieved simply by changing the submatrix of adjacent matrix *A*, denoted as

(6) $$\begin{array}{c} G_{\text{MD}}=\left[\begin{array}{cc} G_{\text{HS}} & G_{S} \end{array}\right],\\ G_{\text{DD}}=\left[\begin{array}{cc} D_{\text{PHS}} & 0\\ 0 & D_{\text{PS}} \end{array}\right], \end{array}$$

where *D*_PHS_ and *D*_PS_ represent human disease and disease of other species, respectively. *G*_HS_ and *G*_*S*_ are microRNA-disease association of human and other species, respectively. When we conduct an experiment on human, set *D*_PS_ = 0 and *G*_*S*_ = 0.

### 3.2. CATAPULT

CATAPULT is a supervised learning method. General supervised learning methods need positive examples and negative examples. However, for microRNA-disease association, there is a lack of negative examples. Positive associations can be checked through existing methods, but there is not a method to prove negative associations. Because negative associations are seldom proven, we processed the problem by treating all nonpositive association node pairs as unlabeled because previous studies have shown that most unlabeled pairs have a negative association [55].

A study by Mordelet and Vert [61] used the bagging technique to obtain an aggregate classifier based on positive examples and unlabeled examples. CATAPULT uses a biased support vector machine (SVM) to classify microRNA-disease pairs. Hence, CATAPULT uses a bagging algorithm to train biased SVM. In CATAPULT, unlabeled samples are randomly selected from the set of all unlabeled examples and a classifier is used to train the selected unlabeled samples as negative examples and positive examples. The features of microRNA-disease pairs are obtained from hybrid walks through the heterogeneous network. To some extent, bagging could reduce the variance in the classifier. The variance is caused by randomly selecting negative examples. *R* is the set of randomly selected negative microRNA-disease pairs and *N*− is the number of set *R*. *T* is the set of positive microRNA-disease pairs and *N*+ is the number of set *T*. *U* denotes all the unlabeled microRNA-disease pairs. The biased SVM means that we assign a penalty, *k*−, for false positives and a larger penalty, *k*+, for false negatives. Detail of the CATAPULT algorithm is displayed in the following part. To train a biased SVM, CATAPULT uses formula (7) based on the known positive examples *T* and randomly selected negative examples *R* to obtain a biased SVM. *ξ*_*i*_ denotes the distance of example *i* from a boundary and SVM gives the example *i* corresponding penalty. 〈*θ*_*t*_, Φ(*x*)〉 denotes the function score for iteration *t*, where *θ*_*t*_ is the normal to the hyper plane at the *t*th iteration and Φ(*x*) is the feature vector of example *x*. Besides, the feature vector of example *x* is the feature vector of the microRNA-disease pair. In our experiment, we assign 1 to *k*− and 30 to *N*− [36].

*CATAPULT Algorithm.*

INIT

For *t* = 1,2, 3,…, *N*−:

(1) Select the set *R* of size *N*− from *U* as negative examples.
(2) Train a classifier based on positive examples *T* and negative examples
  (7)  min⁡θ′∈Rd         12θ′2+k−∑i∈Rξi+k+∑i∈Tξi subject  to  ξi≥0, ∀i∈R∪T, subject  to  Φxi,θ′≥1−ξi, ∀i∈T,subject  to  −Φxi,θ′≥1−ξi, ∀i∈R.
(3) For any* update*:
  (8) $$\begin{array}{c} \;nx⟵nx+1\\ \;s\left(x\right)⟵s\left(x\right)+\langle \theta_{t},\Phi \left(x\right)\rangle \\ \text{return}\;\;\;s\left(x\right)⟵\frac{s\left(x\right)}{n\left(x\right)},\;\forall x\in U. \end{array}$$

## 4. Implementation

### 4.1. Results

The KATZ and CATAPULT methods were applied to the 242 known microRNA-disease associations to infer potential microRNA-disease associations. First, we mainly verified microRNA-disease associations. The set of 242 known microRNA-disease associations is regarded as the “gold standard” data and was used to evaluate the performance of KATZ and CATAPULT methods in the leave-one-out and 3-fold cross-validation experiment and training dataset in the comprehensive prediction [62]. To compare our methods with MBSI, PBSI, and NetCBI, we carried out leave-one-out cross-validation on microRNA-disease associations using KATZ and CATAPULT methods. Furthermore, we carried out the 3-fold cross-validation to make sure that the outperformance of KATZ and CATAPULT is solid. For the leave-one-out cross-validation, each of the 242 known microRNA-disease associations is left out once in turn as the testing case. For the 3-fold cross-validation, the dataset containing 242 known microRNA-disease associations is divided into three parts, which is turned to act as testing. We ranked all microRNA-disease associations according to the scores obtained from KATZ and CATAPULT results.

**Figure 4:**
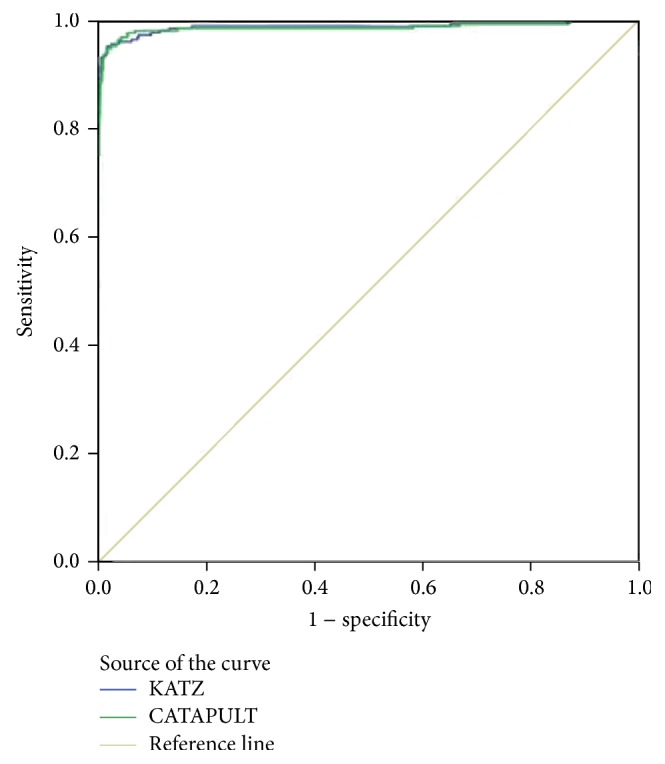
ROC curves of KATZ and CATAPULT methods by leave-one-out cross-validation.

We used a receiver operating characteristic (ROC) curve to evaluate the effect of the method. Varying the threshold plots a ROC curve, and the numeric representation of a ROC curve is the area under the curve (AUC). If we could not compare which method was best from the ROC curve, we could compare the AUC. In the experiment of leave-one-out cross-validation, KATZ and CATAPULT were tested on the 242 known microRNA-disease associations and AUC values 98.9% and 98.8% for KATZ and CATAPULT were achieved.  Figure 4 is the corresponding ROC curve of KATZ and CATAPULT methods. This indicates that our methods have great potential to infer new microRNA-associations.

For the leave-one-out cross-validation, we carry out one loop for each known microRNA-disease association. In each loop, we hide a microRNA-disease association in the known association group and run KATZ and CATAPULT methods on the remaining associations repeating 242 times to ensure that each known microRNA-disease association is hidden exactly once. In each loop, we order the 5080 diseases for the microRNAs, which is the hidden association. We rule that if the disease that is the hidden association has the highest *k* value, then prediction is true. The principle behind this rule is that the method is better if it can predict the true microRNA-disease association with higher probability.  Table 3 shows the distribution of diseases on the basis of the number of microRNAs.  Figure 6 presents the result of prediction hidden microRNA-disease associations. The *x*-axis is the threshold *k* and the *y*-axis is the amount of true prediction.  Figure 6 shows the results for KATZ and CATAPULT.

**Figure 5:**
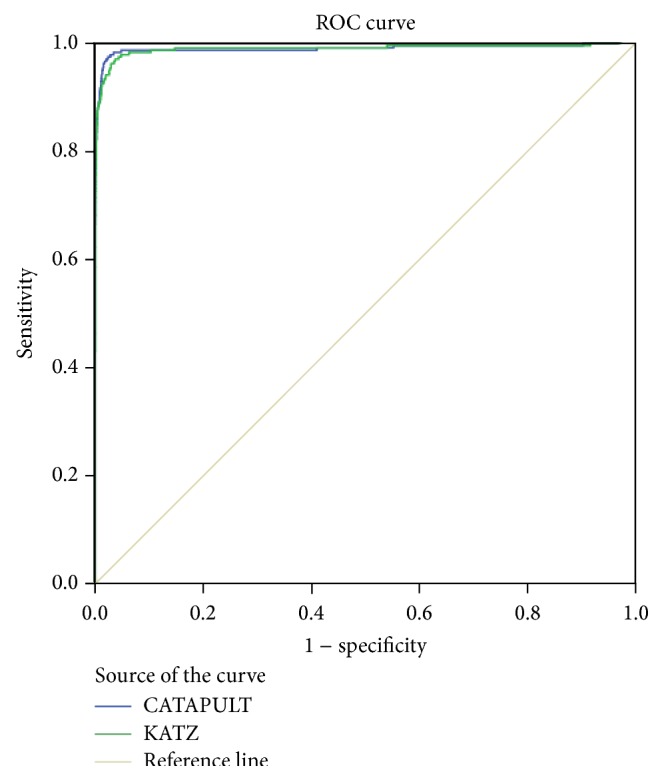
ROC curves of KATZ and CATAPULT methods by 3-fold cross-validation.

**Figure 6:**
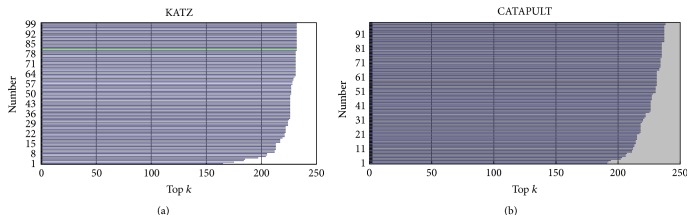
Recovery of microRNA-disease associations with respect to disease rank under leave-one-out cross-validation.

In the experiment of 3-fold cross-validation, KATZ and CATAPULT were tested on the 242 known microRNA-disease associations and AUC values 98.4% and 98.3% for KATZ and CATAPULT were achieved.  Figure 5 shows AUC values of KATZ and CATAPULT methods. The cross-validation results prove that the outperformance is solid.

### 4.2. Evaluation

To confirm the strength of our methods, we compared them with MBSI, PBSI, and NetCBI. MBSI and PBSI both work on the basis of recommendation. However, MBSI takes full advantage of microRNAs similarity. This means that if association between a microRNA and a disease has been validated, then other similar microRNAs would be recommended to the disease. The drawback of MBSI is that it overlooks disease-disease associations. In contrast, PBSI takes full advantage of disease similarities but overlooks the microRNA-microRNA associations. NetCBI considers both associations. The basic idea of NetCBI is ranking. Suppose a microRNA and a disease are linked; if a microRNA is ranked top by querying the microRNAs and a disease is ranked top by querying the diseases, then it rules that associations exist between top-ranking microRNAs and top-ranking diseases.

We used leave-one-out cross-validation to compare our methods with previous methods based on the same datasets.  Table 4 shows the comparative results and our methods are clearly better at predicting microRNA-disease associations than the other methods. The assessment criteria that we used were ROC and AUC. AUC and ROC are the measure of the standard classifier model which is good or bad. ROC presents the evaluation criteria in a visual form, and the AUC value is the area under the ROC curve. Our methods yield 98.9% and 98.8%, which are better than MBSI (74.83%), PBSI (54.02%), and NetCBI (80.66%).

We verify the top 10 predicted associations, which were not identified in our microRNA-disease association dataset. However, the latest online databases provide the evidence. The online databases that we referenced were OMIM, HMDD, and miR2Disease. Tables 5 and 6 show the prediction results by KATZ and CATAPULT. Each predicted association is confirmed by one of the three databases.

## 5. Conclusions

Identifying microRNA-disease associations is an important part of understanding disease mechanisms. Although experimental methods can identity microRNA-disease associations, they are time-consuming and expensive. Hence, efficient methods to identity microRNA-disease associations are desired.

We introduce KATZ and CATAPULT methods for predicting microRNA-disease associations. KATZ succeeds in processing social network links to achieve prediction, which is a different strategy to other methods, such as PBSI and MBSI. The KATZ method uses the entire heterogeneous network, including microRNA-microRNA association, microRNA-disease association, and disease-disease association networks. CATAPULT is a supervised learning method and uses a biased SVM. KATZ and CATAPULT significantly outperform other prediction microRNA-disease association methods, assessed by the leave-one-out and 3-fold cross-validation evaluation strategy. The potential microRNA-disease association predicted by KATZ and CATAPULT will facilitate biological experiments, which identify the true associations between microRNAs and diseases. The KATZ uses the simple measure on the heterogeneous network to predict the potential microRNA-disease associations. KATZ's performance is relatively poor on the sparse known associations.

Although our methods perform well, better methods would be proposed to predict microRNA-disease associations. There are many features of microRNAs and diseases that are not used to help predict microRNA-disease associations, such as gene ontology and the external manifestations of disease. With the use of more factors in prediction methods and the emergence of new relevant data, the prediction of microRNA-disease association will further advance. Ultimately this will help the medical treatment of disease.

## Figures and Tables

**Table 1:** Distribution of the three datasets.

Dataset	Matrix	Similarity score >0
MicroRNA-microRNA association dataset	271 × 271	56289
Disease-disease association dataset	5080 × 5080	20285172
MicroRNA-disease association dataset	271 × 5080	242

**Table 2:** Statistical data for the bipartite graph of the microRNA-disease association network.

Title	Number
MicroRNAs	271
Diseases	5080
Known-associating microRNAs	99
Known-associating diseases	51
Known-associations	242
Average number of microRNA degrees	2.44
Average number of disease degrees	4.75

**Table 3:** Distribution of diseases on the basis of microRNAs.

Number of microRNAs	0	1	2	3	4	5	6	8	9	10	12	15	20	24	27
Number of diseases	5029	16	10	6	3	3	2	4	1	1	1	1	1	1	1

**Table 4:** Comparison of different prediction methods based on AUC values.

Method	MBSI	PBSI	NetCBI	KATZ	CATAPULT
AUC	74.83%	54.02%	80.66%	98.9%	98.8%

**Table 5:** Top 10 newly predicted microRNA-disease associations by KATZ.

Rank	MicroRNA	OMIM disease ID	Disease	Source
1	hsa-let-7i	211980	Lung cancer	HMDD
2	hsa-let-7d	114480	Breast cancer	HMDD
3	hsa-mir-145	211980	Lung cancer	HMDD
4	hsa-mir-18a	114480	Breast cancer	HMDD
5	hsa-mir-145	114480	Breast cancer	HMDD
6	hsa-mir-106b	114480	Breast cancer	HMDD
7	hsa-let-7e	114480	Breast cancer	HMDD
8	hsa-let-7b	114480	Breast cancer	HMDD
9	hsa-mir-19a	114480	Breast cancer	HMDD
10	hsa-mir-125a	114480	Breast cancer	HMDD

**Table 6:** Top 10 newly predicted microRNA-disease associations by CATAPULT.

Rank	MicroRNA	OMIM disease ID	Disease	Source
1	hsa-let-7a	176807	Prostate cancer	miR2Disease
2	hsa-mir-34a	114480	Breast cancer	HMDD
3	hsa-mir-21	211980	Lung cancer	HMDD
4	hsa-let-7c	114480	Breast cancer	HMDD
5	hsa-mir-19a	114480	Breast cancer	HMDD
6	hsa-let-7a	151400	Chronic lymphocytic leukemia	miR2Disease
7	hsa-mir-29b	114480	Breast cancer	miR2Disease
8	hsa-mir-146a	211980	Lung cancer	HMDD
9	hsa-mir-155	211980	Lung cancer	HMDD
10	hsa-let-7c	114550	Hepatocellular carcinoma	miR2Disease
